# Unimodal Latitudinal Pattern of Land-Snail Species Richness across Northern Eurasian Lowlands

**DOI:** 10.1371/journal.pone.0104035

**Published:** 2014-08-04

**Authors:** Michal Horsák, Milan Chytrý

**Affiliations:** Department of Botany and Zoology, Masaryk University, Brno, Czech Republic; University of Sydney, Australia

## Abstract

Large-scale patterns of species richness and their causes are still poorly understood for most terrestrial invertebrates, although invertebrates can add important insights into the mechanisms that generate regional and global biodiversity patterns. Here we explore the general plausibility of the climate-based “water-energy dynamics” hypothesis using the latitudinal pattern of land-snail species richness across extensive topographically homogeneous lowlands of northern Eurasia. We established a 1480-km long latitudinal transect across the Western Siberian Plain (Russia) from the Russia-Kazakhstan border (54.5°N) to the Arctic Ocean (67.5°N), crossing eight latitudinal vegetation zones: steppe, forest-steppe, subtaiga, southern, middle and northern taiga, forest-tundra, and tundra. We sampled snails in forests and open habitats each half-degree of latitude and used generalized linear models to relate snail species richness to climatic variables and soil calcium content measured in situ. Contrary to the classical prediction of latitudinal biodiversity decrease, we found a striking unimodal pattern of snail species richness peaking in the subtaiga and southern-taiga zones between 57 and 59°N. The main south-to-north interchange of the two principal diversity constraints, i.e. drought stress vs. cold stress, explained most of the variance in the latitudinal diversity pattern. Water balance, calculated as annual precipitation minus potential evapotranspiration, was a single variable that could explain 81.7% of the variance in species richness. Our data suggest that the “water-energy dynamics” hypothesis can apply not only at the global scale but also at subcontinental scales of higher latitudes, as water availability was found to be the primary limiting factor also in this extratropical region with summer-warm and dry climate. A narrow zone with a sharp south-to-north switch in the two main diversity constraints seems to constitute the dominant and general pattern of terrestrial diversity across a large part of northern Eurasia, resulting in a subcontinental diversity hotspot of various taxa in this zone.

## Introduction

The overall decline in species diversity with increasing latitude is one of the most prominent features of the natural world (e.g. [1], [2], [3], [4]), but the causes of this gradient remain insufficiently explained in spite of many hypotheses proposed and extensive discussions (e.g. [5], [6], [7]). Previous research was biased to some taxa and regions, and little evidence still exists for terrestrial invertebrates (e.g. [2], [8]). While recent studies stress the greater importance of climate than geographical location (e.g. [9], [10]), the mechanisms by which latitudinal variation in climate determines species numbers are still poorly understood ([7], [11]). As latitude correlates with a number of interacting and inter-correlated environmental gradients (e.g. temperature, precipitation, seasonality, evapotranspiration), direct tests of the hypotheses are difficult and can be controversial ([2]). Although most studies have recognized the “classical” pattern of decreasing species richness towards the poles, both positive and unimodal relationships were repeatedly revealed in several taxa (see [2]). These exceptions were found to be scale dependent. Almost all positive relationships were found across small latitudinal extents (<20° latitude), being associated with regional patterns of heterogeneity in local topography, geology, hydrology, or historical factors ([2]). In contrast, unimodal relationships with a mid-latitudinal peak of diversity were all but one found across broad extents (>20° latitude). The examples came mostly from studies of terrestrial insects (e.g. [12], [13]), but there is also evidence for aquatic invertebrates, herbs, marine and terrestrial birds, and mammals (e.g. [11], [14]). Several explanations were suggested, e.g. mid-latitudinal peak in host density ([15]), mid-domain effect ([13]), habitat specificity ([12]), topographical heterogeneity ([16]) or an increase in resources ([14]). Although the mechanism underlying such unimodal patterns remain poorly known, hardly any doubt exists that climate influences large-scale patterns of species richness. The climatically based “energy hypothesis” has been postulated in several versions and received a considerable attention over the last three decades (e.g. [11], [17], [18]).

In this study we focus on land snails, an invertebrate taxon with as yet poorly explored patterns of latitudinal diversity. Land snail diversity across large scales can be climatically controlled by two principal ecological factors, winter temperature and moisture. Many land snail species apparently do not have any cryoprotective chemicals ([19]), which results in poorly evolved cold-hardiness within this taxon ([20]). The original “freezing tolerance” hypothesis of von Humboldt ([21]) predicts that the number of species is reduced at higher latitudes by the inability of many organisms to withstand low winter temperature ([11]). A commonly recognized decline in land snail species richness towards colder climate at higher elevations ([22], [23]) or in low-productive and environmentally harsh systems with a lack of shelters for overwintering ([8], [24]) suggest that exposure to winter frosts can be a dominant factor shaping latitudinal pattern of land snail diversity. However, there is also a limited number of drought-adapted snail species (e.g. [25]), therefore the number of species generally decreases as conditions become drier (e.g. [26]). Independently of winter temperature, water availability may thus represent the second critical factor, especially in drier areas. While temperature generally decreases with latitude, water availability depends on precipitation, which is independent of latitude, being determined by various systems of atmospheric circulation. Liquid-water availability is also determined by the interaction between temperature and precipitation, because in warmer areas more water is lost due to evapotranspiration. Recent global studies of woody plants (e.g. [27], [28]) indicate that most geographical variation in species richness can be attributed to liquid water-energy dynamics, caused by water doing work ([29]). This mechanism can explain co-variation between climate and richness over space and time ([30], [31]).

In this study we ask (1) what is the latitudinal pattern of land-snail diversity across northern Eurasian lowlands, (2) whether it is determined by low-temperature and drought stress, (3) if so, which of these factors is more important, or whether they interact? To answer these questions, we established a latitudinal transect across Western Siberia spanning 13° from the steppe zone through the forest (taiga) zone to tundra. Western Siberia is the most suitable area for such a study in northern Eurasia, because it is flat lowland with very limited topographic and geological heterogeneity and small human impact, factors which might confound the effects of latitude and macroclimate on species richness in mountainous or densely populated regions. Based on the expected limitation of snail species richness by low temperatures and water deficit, we expect low richness in both dry southern areas of the steppe zone and cold northern areas of the tundra zone. Therefore we hypothesize a unimodal species richness pattern with a peak in the forest zone.

## Materials and Methods

### Study area and sites

**Figure 1:**
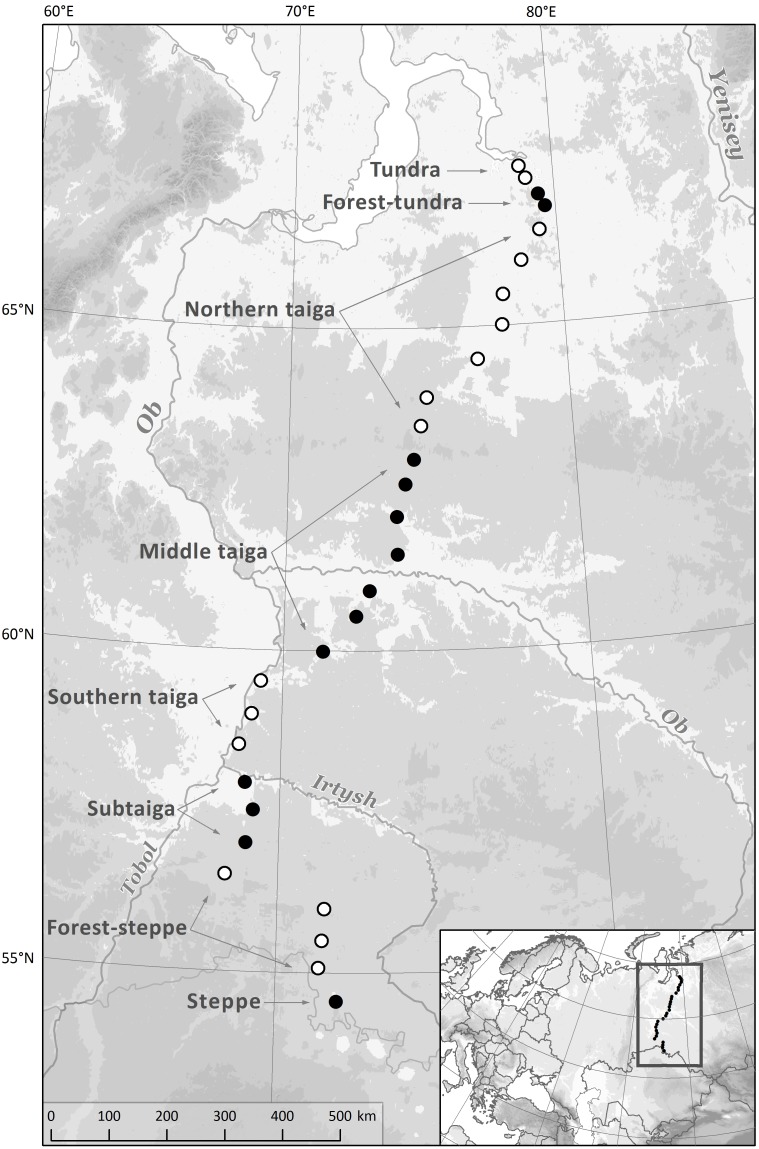
Study area with the position of 29 sampling sites along the transect.

We studied land snail species richness across a latitudinal transect from the northern steppe zone at the Russia-Kazakhstan border SW of the city of Omsk at 54.5°N to the southern tundra zone at the Arctic Ocean coast near the town of Tazovskii at 67.5°N (Fig. 1). The straight-line distance between the southernmost and northernmost site was 1480 km. This transect crossed seven latitudinal vegetation zones ([32]): (**1**) **steppe zone**, dominated by grasses such as *Stipa* and *Festuca*, with rare occurrence of small woodland and shrubland patches in terrain depressions; (**2**) **forest-steppe zone**, consisting of a mosaic of dry to mesic grasslands at the flatland and small open woodlands dominated by birches (*Betula pendula* and *B. pubescens*) and aspen (*Populus tremula*) with many temperate light-demanding species in the herb layer, occurring in shallow terrain depressions; (**3**) **subtaiga zone**, dominated by forests with birches and aspen, with locally admixed fir (*Abies sibirica*) and lime (*Tilia cordata*), and Scots pine (*Pinus sylvestris*) on sandy soils, with a herb layer composed mainly of temperate forest herbs; wet and mesic grasslands occur in scattered patches in a predominantly forested landscape; (**4**) **southern taiga zone**, with the same trees as in the subtaiga zone, but with an additional occurrence of spruce (*Picea obovata*) and Siberian pine (*Pinus sibirica*); grasslands disappear from the landscape, and patches of deep bogs with open low growing stands of Scots pine appear, (**5**) **middle taiga zone**, consisting of a mosaic of coniferous forests with Siberian pine and spruce (on loamy soils) or Scots pine (on sandy soils), frequent occurrence of birch (*Betula pubescens*) and extensive deep bogs; the herb layer of forests is dominated by boreal dwarf shrubs and herbs, bryophytes and lichens, (**6**) **northern taiga zone**, a mosaic of forests with Siberian pine, Scots pine, larch (*Larix sibirica*), spruce and birch (*Betula pubescens*) on permafrost, and shallow bogs including palsas, (**7**) **forest-tundra zone**, semi-open landscape with patches of larch woodlands, extensive stands of dwarf birch (*Betula nana*), grasslands, mires and willow scrub at wet sites; (**8**) **tundra zone**, a treeless landscape with herbaceous and dwarf shrub vegetation, dwarf birch stands, mires and willow scrub.

The whole transect run across the flatland of the West Siberian Plain, ranging in altitude from 148 m (in the south) to 4 m a.s.l. (in the north). Soils were loamy from the steppe zone to the south of the middle taiga zone, and also in the forest-tundra and tundra zones. In contrast, across most of the middle and northern taiga zones soils were predominantly sandy. According to the WorldClim dataset ([33]), mean January temperature ranged from –17°C (in the south) to –26°C (in the north) and mean July temperature from 20°C to 13°C. Annual precipitation ranged from 360 mm (in the south) to 570 mm (in the middle taiga zone).

Sampling sites (n = 29) were placed systematically along the transect, with one site each half degree of latitude. Only in the northernmost part, four sites were spaced by a quarter degree in order to capture the rather narrow zone of forest-tundra. Disturbed sites such as arable land, areas affected by oil or gas extraction or roadsides were avoided. Large river floodplains were avoided too in order to focus on zonal habitats. In some cases, it was necessary to shift the sampling site slightly to the north or south from the target latitude to avoid disturbed sites, floodplains or to sample both forest and open habitats at the same site. However, these shifts were never larger than 5 minutes of latitude.

### Species sampling

At each site we randomly chose three squared sampling plots of 100 m^2^ representing locally predominant habitat types at the site. Because at most sites overall habitat heterogeneity was very low, represented by few homogeneous habitats of large spatial extent, the three plots were sufficient to cover the main habitat heterogeneity. At some sites we had enough time to sample one or two additional plots, which resulted in four and five plots sampled at seven and two sites, respectively (Table 1). This gave a total of 98 plots investigated. In all plots snails were sampled by a single person (M. Horsák). To record as complete inventory of snail assemblage of each plot as possible, various sampling techniques were applied depending on the habitat type investigated. At non-wetland habitats, snails were carefully searched by eye in all microhabitats of the plot for one hour. At species-poor sites (e.g. dry steppes on loess) the sampling was stopped when all recorded species were represented by more than two individuals and at the same time no other species was found for ca. 15 minutes (but sampling was never shorter than 30 minutes). The same stopping rules were used in richer habitats, where sampling took longer time, but not longer than another 30 minutes (in cases of the richest forest plots in the southern taiga). In damper habitat types, i.e. mires, fens and wet tundra, a 12 l sample of the top layer of the ground surface including topsoil, litter, bryophytes and herbaceous vegetation was collected and processed in the field using the wet sieving method ([34]). All recorded shells and live shelled snails were collected and kept dry. Few recorded individuals of slugs were preserved in 70% ethanol. Samples were identified under a dissecting microscope in the laboratory using available identification literature ([35], [36], [37], [38]) and the first author’s comparative collection of Siberian samples. Nomenclature mainly follows [38], but [35] and [37] were used for species of non-European distribution. We declare that no permission was needed as the samples were not collected on privately owned or protected lands and no legally protected species were sampled.

**Table 1:** List of all land-snail species recorded in 98 plots at 29 sites on a latitudinal transect across Western Siberia.

Species/Latitude (°N)	54.5	55.0	55.5	56.0	56.5	57.0	57.5	58.0	58.5	59.0	59.5	60.0	60.5	61.0	61.5	62.0	62.5	63.0	63.5	64.0	64.5	65.0	65.5	66.0	66.5	66.75	67.0	67.25	67.5
*Vallonia pulchella* (Müller)	+	**+**	**+**	**+**	**+**	+	.	.	**.**	**.**	**.**	.	.	.	.	.	.	.	**.**	**.**	**.**	**.**	**.**	**.**	**.**	.	.	**.**	**.**
*Vallonia costata* (Müller)	+	**+**	**+**	**+**	**+**	+	+	+	**+**	**.**	**.**	.	.	.	.	.	.	.	**.**	**.**	**.**	**.**	**.**	**.**	**.**	.	.	**.**	**.**
*Cochlicopa lubricella* (Porro)	+	**+**	**+**	**+**	**+**	+	+	+	**+**	**+**	**.**	.	.	.	.	.	.	.	**.**	**.**	**.**	**.**	**.**	**.**	**.**	.	.	**.**	**.**
*Nesovitrea hammonis* (Ström)	+	**.**	**+**	**.**	**+**	.	+	+	**+**	**+**	**+**	+	+	+	+	.	.	.	**+**	**.**	**+**	**.**	**.**	**.**	**+**	+	+	**.**	**.**
*Succinella oblonga* (Draparnaud)	.	**+**	**.**	**.**	**.**	.	.	.	**.**	**.**	**.**	.	.	.	.	.	.	.	**.**	**.**	**.**	**.**	**.**	**.**	**.**	.	.	**.**	**.**
*Deroceras altaicum* (Simroth)	.	**+**	**+**	**.**	**+**	.	.	.	**.**	**.**	**.**	.	.	.	.	.	.	.	**.**	**.**	**.**	**.**	**.**	**.**	**.**	.	.	**.**	**.**
*Zonitoides nitidus* (Müller)	.	**+**	**+**	**.**	**.**	+	.	.	**.**	**.**	**.**	+	.	.	.	.	.	.	**.**	**.**	**.**	**.**	**.**	**.**	**.**	.	.	**.**	**.**
*Punctum pygmaeum* (Draparnaud)	.	**+**	**+**	**+**	**+**	+	+	+	**+**	**+**	**+**	.	.	.	.	.	.	.	**.**	**.**	**.**	**.**	**.**	**.**	**.**	.	.	**.**	**.**
*Euconulus fulvus* (Müller)	.	**+**	**+**	**+**	**+**	+	+	+	**+**	**+**	**+**	+	+	+	+	+	+	.	**+**	**.**	**+**	**+**	**+**	**+**	**+**	+	+	**+**	**.**
*Vertigo antivertigo* (Draparnaud)	.	**.**	**+**	**.**	**.**	+	.	.	**.**	**.**	**.**	.	.	.	.	.	.	.	**.**	**.**	**.**	**.**	**.**	**.**	**.**	.	.	**.**	**.**
*Vertigo pygmaea* (Draparnaud)	.	**.**	**+**	**.**	**+**	+	+	.	**.**	**.**	**.**	.	.	.	.	.	.	.	**.**	**.**	**.**	**.**	**.**	**.**	**.**	.	.	**.**	**.**
*Euconulus praticola* (Reinhardt)	.	**.**	**+**	**+**	**.**	+	.	.	**.**	**.**	**.**	+	.	.	.	.	.	+	**.**	**.**	**.**	**.**	**.**	**.**	**+**	.	.	**.**	**.**
*Vitrina pellucida* (Müller)	.	**.**	**.**	**+**	**+**	+	+	+	**+**	**.**	**.**	.	.	.	.	.	.	.	**.**	**.**	**.**	**.**	**.**	**.**	**.**	.	.	**.**	**.**
*Fruticicola schrenckii* (Middendorff)	.	**.**	**.**	**.**	**+**	+	+	.	**+**	**.**	**.**	.	+	.	.	.	.	.	**.**	**.**	**.**	**.**	**.**	**.**	**.**	.	.	**.**	**.**
*Discus ruderatus* (Hartmann)	.	**.**	**.**	**.**	**+**	.	+	+	**+**	**+**	**+**	.	+	.	.	.	.	.	**+**	**.**	**.**	**.**	**.**	**.**	**.**	.	.	**.**	**.**
*Carychium minimum* Müller	.	**.**	**.**	**.**	**.**	+	.	.	**.**	**.**	**.**	.	.	.	.	.	.	.	**.**	**.**	**.**	**.**	**.**	**.**	**.**	.	.	**.**	**.**
*Oxyloma elegans* (Risso)	.	**.**	**.**	**.**	**.**	+	.	.	**.**	**.**	**.**	.	.	.	.	.	.	.	**.**	**.**	**.**	**.**	**.**	**.**	**.**	.	.	**.**	**.**
*Vertigo angustior* Jeffreys	.	**.**	**.**	**.**	**.**	+	.	.	**.**	**.**	**.**	.	.	.	.	.	.	.	**.**	**.**	**.**	**.**	**.**	**.**	**.**	.	.	**.**	**.**
*Vertigo substriata* (Jeffreys)	.	**.**	**.**	**.**	**.**	+	.	.	**+**	**+**	**+**	.	.	.	.	.	.	.	**.**	**.**	**.**	**.**	**.**	**.**	**.**	.	.	**.**	**.**
*Columella edentula* (Draparnaud)	.	**.**	**.**	**.**	**.**	+	+	.	**+**	**+**	**+**	.	+	.	.	.	.	.	**.**	**.**	**.**	**.**	**.**	**.**	**.**	.	.	**.**	**.**
*Vertigo pusilla* Müller	.	**.**	**.**	**.**	**.**	.	+	.	**+**	**.**	**.**	.	.	.	.	.	.	.	**.**	**.**	**.**	**.**	**.**	**.**	**.**	.	.	**.**	**.**
*Cochlicopa lubrica* (Müller)	.	**.**	**.**	**.**	**.**	.	+	.	**+**	**+**	**.**	.	.	.	.	.	.	.	**.**	**.**	**.**	**.**	**.**	**.**	**.**	.	.	**.**	**.**
*Succinea putris* (Linné)	.	**.**	**.**	**.**	**.**	.	+	.	**.**	**.**	**.**	+	+	.	.	.	.	.	**.**	**.**	**.**	**.**	**.**	**.**	**+**	.	.	**.**	**.**
*Nesovitrea petronella* (Pfeiffer)	.	**.**	**.**	**.**	**.**	.	+	+	**+**	**+**	**+**	+	+	+	.	.	.	+	**+**	**.**	**.**	**.**	**.**	**.**	**.**	.	.	**.**	**.**
*Vertigo ronnebyensis* (Westerlund)	.	**.**	**.**	**.**	**.**	.	+	.	**+**	**+**	**+**	+	+	+	+	.	.	.	**+**	**.**	**.**	**.**	**.**	**.**	**+**	.	.	**.**	**.**
*Arion fuscus* (Müller)	.	**.**	**.**	**.**	**.**	.	.	+	**.**	**.**	**.**	.	.	.	.	.	.	.	**.**	**.**	**.**	**.**	**.**	**.**	**.**	.	.	**.**	**.**
*Vertigo* aff. *gouldi* Binney	.	**.**	**.**	**.**	**.**	.	.	.	**+**	**.**	**.**	.	.	.	.	.	.	.	**.**	**.**	**.**	**.**	**.**	**.**	**.**	.	.	**.**	**.**
*Vertigo modesta* aff. *hoppii* (Möller)	.	**.**	**.**	**.**	**.**	.	.	.	**.**	**.**	**.**	+	.	.	.	.	.	.	**.**	**.**	**.**	**.**	**.**	**.**	**.**	+	.	**+**	**.**
*Deroceras laeve* (Müller)	.	**.**	**.**	**.**	**.**	.	.	.	**.**	**.**	**.**	.	+	.	.	.	.	.	**.**	**.**	**.**	**.**	**.**	**.**	**.**	.	.	**.**	**.**
*Zoogenetes harpa* (Say)	.	**.**	**.**	**.**	**.**	.	.	.	**.**	**.**	**.**	.	.	+	.	.	.	.	**.**	**.**	**.**	**.**	**.**	**.**	**.**	.	+	**.**	**.**
*Vertigo lilljeborgi* (Westerlund)	.	**.**	**.**	**.**	**.**	.	.	.	**.**	**.**	**.**	.	.	.	.	.	.	.	**.**	**.**	**.**	**.**	**.**	**.**	**+**	.	.	**.**	**.**
* *Vertigo extima* (Westerlund)	.	**.**	**.**	**.**	**.**	.	.	.	**.**	**.**	**.**	.	.	.	.	.	.	.	**.**	**.**	**.**	**.**	**.**	**.**	**.**	.	(+)	**.**	**.**
Total no. of plots	5	**4**	**3**	**4**	**3**	4	3	3	**3**	**3**	**3**	3	4	3	3	4	3	4	**5**	**3**	**3**	**4**	**3**	**3**	**3**	3	3	**3**	**3**
Total no. of species	4	**8**	**11**	**7**	**11**	16	15	9	**15**	**10**	**8**	8	9	5	3	1	1	2	**5**	**0**	**2**	**1**	**1**	**1**	**6**	3	3	**2**	**0**

*this species was recorded at the site but not in the studied plots.

Individual zones (from left to right): steppe, forest-steppe, subtaiga, southern taiga, middle taiga, northern taiga, forest-tundra, and tundra, indicated using alternating bold style. Presence of a species is marked by crosses; species are ordered based on their first finding along the transect from south to north.

### Explanatory variables

For each site, annual precipitation sum and mean January and July temperature were obtained from the WorldClim database ([33], www.worldclim.org) using the ArcGIS 8.3 program (www.esri.com). Annual potential evapotranspiration, calculated from the WorldClim data, was obtained from the website of CGIAR-CSI Consortium for Spatial Information (http://csi.cgiar.org/Aridity/, [39]). We also expressed water balance as the difference between annual precipitation sum and annual potential evapotranspiration ([40]). As land snail species richness and composition is tightly related to calcium availability (e.g. [41]), we also measured calcium (Ca) content in topsoil (or in peat in mires). Although the amount of calcium is strongly correlated with climate due to higher cation leaching in wetter conditions, any heterogeneity in local geology and vegetation cover ([42]) can have positive effects on snail species richness. Therefore we collected soil samples from the mineral topsoil horizon at a depth of 5–10 cm in four places within each plot. These four subsamples were mixed and sieved at a mesh size of 1 mm. Available calcium was extracted from the sieved soil using the Mehlich III method (strong acid extraction with ion complex) and determined by atomic absorption spectrophotometry (AAS 933 Plus, GBC Scientific Equipment, Melbourne, Australia). The analysis was done by AgroLab, Troubsko, Czech Republic according to the methods described by [43].

### Statistical analyses

All basic data used in the analyses and the graphs are provided in Table S1. Correlations among explanatory variables were inspected graphically (Figs. S1 and S2) and quantified using the Spearman correlation coefficient. As tight correlations were found among mean annual temperature, mean July and January temperature (Fig. S1), we used only January temperature as the most ecologically relevant measure of temperature for land snails due to a poorly evolved cold-hardiness within this taxon ([20]).

For each of 29 sampling sites we calculated mean number of species recorded at three to five sampling plots. As the mean number of species at a site tightly correlated with the total number of species recorded at all plots of a site (r_S_ = 0.98, P<0.001), we used only mean numbers (for correlations see Fig. S2). As the majority of temperate and boreal land snail diversity is confined to forest environments ([36], [37]), we also counted total and mean numbers of species recorded at forest and open-habitat plots separately. Relationships between the numbers of species recorded at each of 29 sites and explanatory variables were explored only graphically as there were no systematic trends except for water balance. Therefore, we modelled only the response of mean numbers of species recorded at each site to water balance using a generalized linear model with a Poisson error structure (GLM-p). To correct for under- or over-dispersion, we used a quasi-GLM-p with the variance given by *φ* × *µ*, where *φ* is the dispersion parameter and *µ* is the mean. Both linear and quadratic terms were included into the model and their significance was tested using the *F*-test. The final model was inspected using the distribution of residuals and standardized residuals against predicted values, and Cook’s distances ([44]). The same procedures were used to construct the most parsimonious model and to find a set of uncorrelated significant predictors. The minimal adequate model, starting with a full model that included both linear and quadratic terms of all predictors (except water balance that combines precipitation and evapotranspiration) and all two-way interactions, was established using a stepwise deletion procedure based on *F*-tests. To visualize changes of diversity with the most important climatic and soil variables, the changes of these variables with latitude were expressed using the locally-weighted polynomial regression. The obtained regression lines were overlaid with the diversity changes in a single plot. The regression was calculated using the function “*lowess*” ([45]). All graphics and calculations were performed using the R program ([46]).

## Results

**Figure 2:**
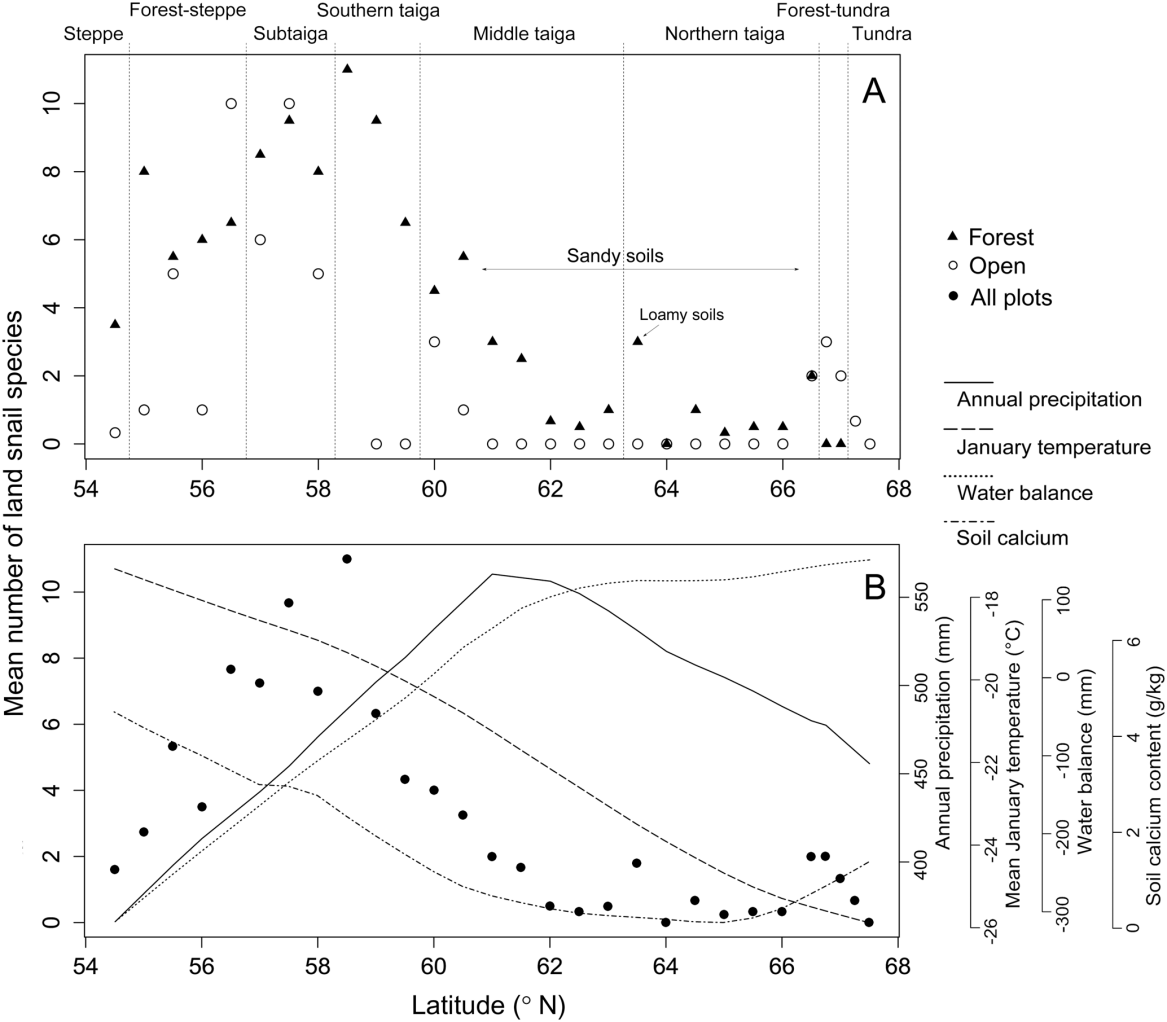
Mean numbers of land snail species recorded in 100 m^2^ plots at 29 sites located along a latitudinal transect in the West Siberian Plain between 54.5° and 67.5°N. **A**, species from forest and open habitats counted separately; **B**, means from all plots at each site shown with patterns of four environmental variables visualized using the locally-weighted polynomial regression.

We found a total of 33 land snail species (Table 1) represented by 2,788 individuals in 98 studied plots. Cumulative numbers of species recorded per site varied from 0 to16 and the average per plot ranged between 0 and 11 (Fig. S2). Forest plots were notably richer in species than open plots at almost all sites except for those in the forest-tundra zone (Fig. 2A). Median numbers of species were 4 and 0 (mean 4.1 and 1.6) for forest and open-habitat plots, respectively. However, the difference in the cumulative lists of species recorded in these two contrasting habitat types was not high, as 28 species were found in 54 forest plots and 22 species in 44 open plots.

**Figure 3:**
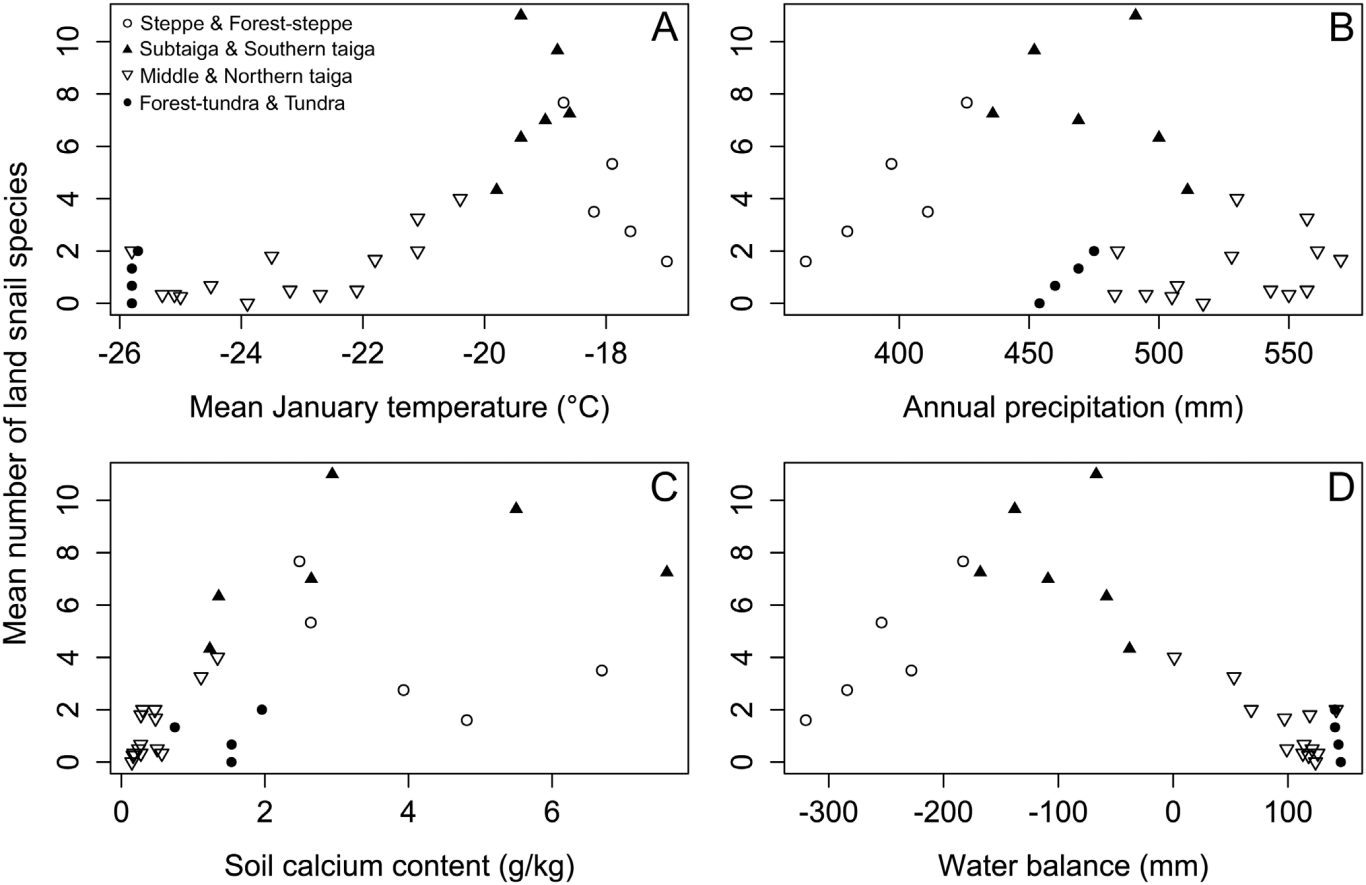
Changes in mean numbers of land snail species in relation to four environmental variables.

We found a pronounced unimodal pattern of species richness along the latitudinal gradient, peaking between 56.5° and 59.0°N (Fig. 2). This pattern was detected for both cumulative and mean number of species as well as for both forest and open habitats (Fig. S2). Taking forest plots separately, the peak was in the subtaiga and southern taiga zones (Fig. 2A). In the southern part of the transect, the number of species increased linearly with precipitation, especially in the steppe and forest-steppe zones, reaching the maximal mean value at the transition between the subtaiga and southern taiga zones (Fig. 3B). After that species richness declined, reflecting the appearance of acidic mires with no snails recorded (Fig. 2A). For forest species, a linear decrease of species richness started at the transition between southern taiga and middle taiga and continued throughout the latter zone (Fig. 2A). The number of species was linearly decreasing with temperature and soil calcium content (Fig. 2B). Harsh conditions, strengthened by the presence of sandy soils, kept the number of species very low from the northern margin of the middle taiga zone across the whole of the northern taiga zone. At the transition to the forest-tundra zone and within this zone an increase in the number of species was found, likely associated with the increase in calcium content in the soils (Figs. 2B and S2). However, in the tundra zone the number of species linearly dropped down to zero.

Water balance was the single variable that expressed a tight relationship with species richness along the whole latitudinal extent (Fig. 3D). Using a generalized linear model we found highly significant quadratic response of the mean number of species to water balance. This single predictor was able to explain 81.7% of the total variance in mean numbers of species (quasi-GLM-p, p<<0.001). The most parsimonious model (with water balance not included, see Methods) consisted of annual precipitation (linear and quadratic term), mean January temperature (linear term), soil calcium content (linear term) and interaction between the quadratic term of annual precipitation and soil calcium. The model explained 87.3% of the total variance in species richness (quasi-GLM-p, p<<0.001).

## Discussion

Our data suggest a climatically controlled unimodal pattern of latitudinal land snail diversity across Western Siberia. Previous reports of unimodal diversity pattern along latitude originated from larger spatial extents ([12], [13], [16]) and were explained by various mechanisms, although climatically related. The pattern observed for land snails in Western Siberia was controlled by two main climatic constraints that were, however, operating at different latitudes: drought stress in the south and cold stress in the north. Hawkins et al. [11] analysed global diversity of terrestrial birds and found that linear association of climate with species richness at continental extents is rather exceptional. The data from the former USSR reported in their study (see Figure six in [11]) suggest the same unimodal pattern as we found for land snail diversity, with a peak in the subtaiga and southern taiga zones. A similar pattern was also shown for amphibians (Figure one in [47]), which seems to be more prominent towards the western Palaearctic. As we observed the same pattern along the studied transect also for vascular plant diversity (unpubl. data), which partly coincides with the coarse-scale map of plant species numbers per 1000 km^2^ ([48]), the climatically driven latitudinal change in diversity seems to be universal for this part of Eurasia. However, the mechanisms shaping these unimodal diversity changes can differ among individual taxa. With a high certainty we can reject Rosenzweig’s ([49]) “habitat heterogeneity” hypothesis, likely explaining a unimodal latitudinal pattern of plant diversity in continental Chile ([16]), as the observed changes in the number of land snail species in Western Siberia occurred on flatland, in absence of any significant topographical variance along the transect (elevation of studied plots along the whole transect varied between 148 and 4 m a.s.l.).

There was no clear relation between the heterogeneity of habitat types in the landscape and local species richness at particular sites, either mean or cumulative. We found southern taiga zone to be the richest in species, although the landscape was dominated by a single habitat type, mixed broadleaf-conifer hemiboreal forest. Among forest assemblages, compositional pattern was nested, with species accumulating towards the southern taiga zone from both latitudinal directions. However, the diversity pattern was notably different between forest and open habitats. Open habitat types changed more dramatically than forest types with latitude, continuously replacing one another along the transect, from dry steppe through mesic and wet grasslands and bogs to herbaceous and shrubby tundra. This constituted a considerable compositional turnover with no shared snail species for the steppe and tundra zones. This difference also explains why the cumulative numbers of species recorded in all forest and open plots were not much different, despite often high differences in the numbers for individual plots.

The observed changes in species richness can possibly result from different colonization histories along the transect. However, there are no obvious geographical barriers for land snails that could hamper species migration as almost all recorded snails belong to minute species known as very effective passive dispersers (e.g. [50], [51]). Further, the whole area was ice-free during the last glacial maximum ([52]), so there was enough time for species to saturate all favourable habitats. Further, almost all recorded species have wide distributions (Holarctic, Palaearctic, Siberian) with ranges extending across the temperate and boreal zones of the continent. Thus, there seems to be no significant imprint of the postglacial colonization history in this part of Eurasia, as known from western and central Europe ([51], [53]).

We observed a sharp drop of species numbers with decreasing water availability in areas of high ambient energy that supports the “water-energy dynamics” hypothesis developed for plant diversity ([27], [29]). As the major predictions of this hypothesis were fulfilled also by the global diversity pattern of terrestrial birds, Hawkins et al. [11] suggested this hypothesis as a unifying hypothesis for terrestrial diversity gradients. It postulates that measures of ambient energy are the best predictors of diversity patterns in high-latitude regions, while water-related variables best explain diversity gradients in the subtropics and tropics, i.e. areas with high energy input ([30]). Our data suggest a broader plausibility of this prediction, as water availability can be the primary limiting factor also in extratropical regions with warm and dry climate. Although the ambient energy was proposed as the ultimate constraint for most terrestrial organisms above ca. 50°N (e.g. [17], [54]), we found the main interchange of the two principal diversity constraints (cold stress vs. drought stress) between 57 and 59°N. It seems to be a dominant and general pattern of diversity across a great part of northern Eurasia, from Eastern Europe to Eastern Siberia (see Figure six in [11]). Water-related decrease in bird diversity from the southern taiga towards the dry steppe zone of Kazakhstan, Uzbekistan and Turkmenistan, occurs between 38°and 58° of latitude. Our transect ended at the northern margin of this zone, but further continuous decrease in diversity towards southern part of this warm and dry region is very likely. Dry steppes or saline habitats in this region typically contain no snails in small plots. The unimodal change in snail species richness, though caused by different limiting factors, is very well described by water balance, a variable describing moisture availability resulting from the counteracting effects of precipitation and evapotranspiration. However, a more complex model incorporating both precipitation and temperature as separate variables, accompanied with soil calcium, was able to explain slightly more variation in species data. Likewise, the balance of temperature and precipitation (included in both linear and quadratic terms) was the best overall predictor of the variation in global amphibian richness ([47]).

The question remains, however, which mechanism causes the decline of species richness from the mixed forests of southern taiga towards the north. Decreasing ambient energy can control diversity by low temperature stress (freezing) or low productivity that translates into lower availability of food and shelters for overwintering at higher latitudes. There is not only a strong collinearity but also a strong positive feedback between these two factors. In ecosystems with very cool winters, distribution of terrestrial snails is constrained by the lack of shelters for overwintering ([8]), which becomes more pronounced where herbaceous biomass production is low ([24]). Considering the known physiological constraints of land snails (e.g. [19]), low temperature stress is a more parsimonious explanation than the limited food availability. Some species frequently inhabiting low-productive steppe ecosystems (e.g. *Vallonia pulchella*) are not able to cope with open tundra habitats of similar productivity levels ([55]). The freezing intolerance hypothesis is also supported by a limited distribution of large-bodied species towards higher altitudes, as smaller species have better supercooling abilities ([20]). Although the freezing hypothesis was repeatedly rejected for terrestrial birds (e.g. [11]), temperature can be more serious constraint for ectotherms, as supported by the analysis of global amphibian diversity ([47]). In land snails particularly, we need to consider collinearity between the poleward decline in temperature and soil calcium content, likely occurring in most regions due to lower evaporation of soil water and consequent higher cation leaching at higher latitudes. Content of soil calcium showed a more complex pattern along our latitudinal transect. The amount of calcium dropped steeply between the southern and middle taiga zones, being very low in most of the middle and northern taiga zones (a zone with predominant sandy soils), but increased again in the forest-tundra and tundra zones (Fig. 2A). As land snail species richness typically increases with increasing availability of calcium ([41], [42], [56], [57]), more acidic conditions at higher latitudes can contribute to the decline in species richness independently of temperature. Although the causality of these two factors cannot be disentangled using our data, we suggest that the main effect is that decreasing species richness at higher latitude is primarily determined by low winter temperature, but it is further modified by low calcium levels, which may cause an additional reduction of the number of species. Although extremely calcium-poor conditions occurred in the middle and northern taiga zone due to the predominance of acidic sandy soils, smaller areas of loamy soils can be found there. At 63.5°N we sampled four adjacent forest plots, two on sandy and two on loamy soils. Loamy-soil plots were richer in both the calcium content (ca. 0.51 g/kg) and the number of species (five and three) in contrast to sandy-soil plots with ca. 0.15 g/kg of soil calcium, which hosted only one or no snail species. Higher content of calcium (ca. 1.5 g/kg) in the forest-tundra and tundra soils in comparison with those in the southern taiga zone (ca. 1.3 g/kg) clearly demonstrates that higher calcium content is necessary, but not sufficient condition for achieving a given level of species richness (Fig. 3C). Climatically driven decline in diversity was notable particularly in forests of the forest-tundra zone, which remained very poor in snails even under more calcium-rich conditions (Fig. 2A). The increase in species richness in open habitats of this zone was associated with the first appearance of the arctic *Vertigo* species (Table 1), recorded in wet willow scrub or fen tundra vegetation. These species belong to the smallest land snails, which are able to survive under very low temperature, contrary to the majority of forest species.

The observed responses of land snail species richness to two largely independent factors, water availability and winter temperature, are important for considerations about future changes of latitudinal diversity patterns induced by future climate change ([58]). As geographical patterns of precipitation and temperature shift rather independently, future shifts in the distribution of species and whole ecosystems can be rather complex. This can possibly result in a disintegration of the most suitable zone of the highest diversity, i.e. subtaiga and southern taiga, which are now both warm and wet. Containing nearly 80% of the total snail species number recorded from the steppe to the tundra zone, these two rather narrow zones represent the main diversity hot-spot in this part of northern Eurasia. In this study, we also provided evidence that no single factor is sufficient to explain the unimodal latitudinal gradient in diversity. This adds to a growing body of evidence that multiple factors vary in concert with latitude and interact to cause the latitudinal gradient of species richness both on global and continental scales (e.g. [2], [10], [11], [27], [47], [59]). Our data extend plausibility of this observation also to smaller spatial extents.

## Supporting Information

Figure S1 Patterns of selected climatic and environmental variables along the studied latitudinal transect and their pairwise relationships. The upper right part shows values of Spearman correlation coefficient.
(TIF)

Figure S2 Changes in land-snail species numbers measured in different habitats and different ways along the studied latitudinal transect and their pairwise relationships. The upper right part shows values of Spearman correlation coefficient.
(TIF)

Table S1 Data used in the analyses with numbers of all recorded land snail species (All Sum) at each of the 29 studied sites, mean numbers of species (All Mean), and means of species recorded at open (Open Mean) and forest (Forest Mean) habitats separately at each site; numbers of open and forest plots sampled is given in the second column (Open/Forest). NA refers to the situations where no open or forest habitats were sampled. Values of all explanatory variables used in the study are also provided.
(XLSX)

## References

[1] Rosenzweig ML (1995) Species diversity in space and time. Cambridge: Cambridge University Press. 436 p.

[2] Willig MR, Kaufmann DM, Stevens RD (2003) Latitudinal gradients of biodiversity: pattern, process, scale and synthesis. Annu Rev Ecol Syst 34: 273–309.

[3] Pimm SL, Brown JH (2004) Domains of diversity. Science 304: 831–833. 15131295 10.1126/science.1095332

[4] Nekola JC (2005) Latitudinal richness, evenness, and shell size gradients in eastern North American land snail communities. Rec West Aust Mus Suppl. 68: 39–51.

[5] Bromham L, Cardillo M (2003) Testing the link between the latitudinal gradient in species richness and rates of molecular evolution. J Evol Biol 16: 200–207. 14635858 10.1046/j.1420-9101.2003.00526.x

[6] Hillebrand H (2004) On the generality of the latitudinal diversity gradient. Amer Nat 163: 192–211. 14970922 10.1086/381004

[7] Cardillo M, Orme CDL, Owens IPF (2005) Testing for latitudinal bias in diversification rates: an example using New World birds. Ecology 86: 2278–2287.

[8] Horsák M, Chytrý M, Axmanová I (2013) Exceptionally poor land snail fauna of central Yakutia (NE Russia): climatic and habitat determinants of species richness. Polar Biol 36: 185–191.

[9] Francis AP, Currie DJ (2003) A globally consistent richness–climate relationship for angiosperms. Amer Nat 161: 523–536. 12776882 10.1086/368223

[10] Field R, Hawkins BA, Cornell HV, Currie DJ, Diniz-Filho AJF, et al (2009) Spatial species-richness gradients across scales: a meta-analysis. J Biogeogr 36: 132–147.

[11] Hawkins BA, Porter EE, Diniz-Filho JAF (2003) Productivity and history as predictors of the latitudinal diversity gradient of terrestrial birds. Ecology 84: 1608–1623.

[12] Davidowitz G, Rosenzweig ML (1998) The latitudinal gradient in species diversity among North American grasshoppers (Acrididae) within a single habitat: a test of the spatial heterogeneity hypothesis. J Biogeogr 25: 553–560.

[13] Skillen EL, Pickering J, Sharkey MJ (2000) Species richness of the Campopleginae and Ichneumoninae (Hymenoptera: Ichneumonidae) along a latitudinal gradient in eastern North America old-growth forests. Environ Entomol 29: 460–466.

[14] Chown SL, Gaston KJ, Williams PH (1998) Global patterns in species richness of pelagic seabirds: the Procellariiformes. Ecography 21: 342–350.

[15] Janzen DH (1981) The peak in North American ichneumonid species richness lies between 38° and 42° North. Ecology 62: 532–537.

[16] Bannister JR, Vidal OJ, Teneb E, Sandoval V (2012) Latitudinal patterns and regionalization of plant diversity along a 4270-km gradient in continental Chile. Austral Ecol 37: 500–509.

[17] Currie DJ (1991) Energy and large-scale patterns of animal and plant-species richness. Amer Nat 137: 27–49.

[18] Mittelbach GG, Steiner CF, Scheiner SM, Gross KL, Reynolds HL, et al (2001) What is the observed relationship between species richness and productivity? Ecology 82: 2381–2396.

[19] Riddle WA (1983) Physiological ecology of snails and slugs. In: Russell-Hunter WD, editor. The Mollusca, Vol. 6: Ecology. London: Academic Press, 431–461.

[20] Ansart A, Vernon P (2003) Cold hardiness in molluscs. Acta Oecol 24: 95–102.

[21] von Humboldt A (1808) Ansichten der Natur, mit wissenschaftlichen Erläuterungen. Tübingen: J. G. Cotta, 474 p.

[22] Cameron RAD, Greenwood JJD (1991) Some montane and forest molluscan faunas from eastern Scotland: effects of altitude, disturbance and isolation. In: Meier-Brook C, editor. Proceedings of the 10th International Malacological Congress. Tübingen: Unitas Malacologica, 437–442.

[23] Horsák M, Cernohorsky N (2008) Mollusc diversity patterns in Central European fens: hotspots and conservation priorities. J Biogeogr 35: 1215–1225.

[24] Schamp B, Horsák M, Hájek M (2010) Deterministic assembly of land snail communities according to species size and diet. J Anim Ecol 79: 803–810. 20345504 10.1111/j.1365-2656.2010.01685.x

[25] Ložek V (1964) Quartärmollusken der Tschechoslowakei. Praha: Nakladatelství Československé akademie věd. 374 p.

[26] Martin K, Sommer M (2004) Relationships between land snail assemblage patterns and soil properties in temperate-humid forest ecosystems. J Biogeogr 31: 531–545.

[27] O’Brien EM (1998) Water–energy dynamics, climate, and prediction of woody plant species richness: an interim general model. J Biogeogr 25: 379–398.

[28] O’Brien EM, Whittaker RJ, Field R (1998) Climate and woody plant diversity in southern Africa: relationships at species, genus and family levels. Ecography 21: 495–509.

[29] O’Brien EM (2006) Biological relativity to water–energy dynamics. J Biogeogr 33: 1868–1888.

[30] Hawkins BA, Field R, Cornell HV, Currie DJ, Guegan JF, et al (2003) Energy, water, and broad-scale geographic patterns of species richness. Ecology 84: 3105–3117.

[31] Field R, O’Brien EM, Whittaker RJ (2005) Global models for predicting woody plant richness from climate: development and evaluation. Ecology 86: 2263–2277.

[32] Walter H (1974) Die Vegetation Osteuropas, Nord- und Zentralasiens. Stuttgart: Gustav Fischer Verlag. 452 p.

[33] Hijmans RJ, Cameron SE, Parra JL, Jones PG, Jarvis A (2005) Very high resolution interpolated climate surfaces for global land areas. Inter J Climatol 25: 1965–1978.

[34] Horsák M (2003) How to sample mollusc communities in mires easily. Malacologica Bohemoslovaca 2: 11–14.

[35] Pilsbry HA (1948) Land Mollusca of North America north of Mexico. Vol. II, part 2. Philadelphia: The Academy of Natural Sciences of Philadelphia. 1113 p.

[36] Kerney MP, Cameron RAD, Jungbluth JH (1983) Die Landschnecken Nord- und Mitteleuropas. Hamburg/Berlin: Parey Verlag. 384 p.

[37] Sysoev A, Schileyko A (2009) Land snails of Russia and adjacent countries. Sofia/Moscow: Pensoft. 312 p.

[38] Horsák M, Juřičková L, Picka J (2013) Molluscs of the Czech and Slovak Republics. Zlín: Kabourek. 264 p.

[39] Trabucco A, Zomer RJ, Bossio DA, van Straaten O, Verchot LV (2008) Climate change mitigation through afforestation/reforestation: A global analysis of hydrologic impacts with four case studies. Agricul Ecosyst Environ 126: 81–97.

[40] Churkina G, Running SW, Schloss AL, the participants of the Potsdam NPP model intercomparison (1999) Comparing global models of terrestrial net primary productivity (NPP): the importance of water availability. Glob Change Biol 5 (Suppl. 1)46–55.

[41] Juřičková L, Horsák M, Cameron R, Hylander K, Míkovcová A, et al (2008) Land snail distribution patterns within a site: the role of different calcium sources. Eur J Soil Biol 44: 172–179.

[42] Wäreborn I (1969) Land molluscs and their environments in an oligotrophic area in southern Sweden. Oikos 20: 461–479.

[43] Zbíral J (1995) [Analysis of plant material. Unified techniques]. Brno: Central Institute for Supervising and Testing in Agriculture. 192 p. [In Czech].

[44] Cook R D (1977) Detection of influential observation in linear regression. Technometrics 19: 15–18.

[45] Cleveland WS (1979) Robust locally weighted regression and smoothing scatterplots. J Amer Stat Assoc 74: 829–836.

[46] R Core Team (2012) R: A language and environment for statistical computing. R Foundation for Statistical Computing, Vienna- URL http://www.R-project.org/.

[47] Buckley LB, Jetz W (2007) Environmental and historical constraints on global patterns of amphibian richness. Proc R Soc Lond B 274: 1167–1173. 10.1098/rspb.2006.0436 PMC2189569 17327208

[48] Malyshev LI (1993) Ecological background of the floristic diversity in northern Asia. Fragm Flor Geobot Suppl. 2(1): 331–342.

[49] Rosenzweig ML (1992) Species diversity gradients: we know more and less than we thought. J Mammal 73: 715–730.

[50] Nekola JC (2009) Big ranges from small packages: North American vertiginids more widespread than thought. The Tentacle 17: 26–27.

[51] Cameron RAD, Pokryszko BM, Horsák M (2010) Land snail faunas in Polish forests: patterns of richness and composition in a post-glacial landscape. Malacologia 53: 77–134.

[52] Ehlers J, Gibbard PL, editors (2004) Quaternary glaciations - extent and chronology, Part III: South America, Asia, Africa, Australasia, Antarctica. Amsterdam: Elsevier. 903 p.

[53] Hausdorf B, Hennig C (2003) Nestedness of northwest European land snail ranges as a consequence of differential immigration from Pleistocene glacial refuges. Oecologia 135: 102–109. 12647109 10.1007/s00442-002-1142-y

[54] Lennon JJ, Greenwood JJD, Turner JRG (2000) Bird diversity and environmental gradients in Britain: a test of the species–energy hypothesis. J Anim Ecol 69: 581–598.

[55] Del Grosso S, Parton W, Stohlgren T, Zheng D, Bachelet D, et al (2008) Global potential net primary production predicted from vegetation class, precipitation, and temperature. Ecology 89: 2117–2126. 18724722 10.1890/07-0850.1

[56] Hylander K, Nilsson C, Jonsson BG, Göthner T (2005) Differences in habitat quality explain nestedness in a land snail metacommunity. Oikos 108: 351–361.

[57] Horsák M (2006) Mollusc community patterns and species response curves along a mineral richness gradient: a case study in fens. J Biogeogr 33: 98–107.

[58] IPCC (2007) Climate change 2007: The physical science basis. Contribution of Working Group I to the Fourth Assessment Report of the Intergovernmental Panel on Climate Change. Cambridge: Cambridge University Press. 582 p.

[59] Willis KJ, Whittaker RJ (2002) Species diversity–scale matters. Science 295: 1245–1248. 11847328 10.1126/science.1067335

