# Supplementary figures and images for: Unimodal Latitudinal Pattern of Land-Snail Species Richness across Northern Eurasian Lowlands
Source: PLoS One. 2014 Aug 4;9(8):e104035. doi: 10.1371/journal.pone.0104035 (PMC4121278; doi:10.1371/journal.pone.0104035)

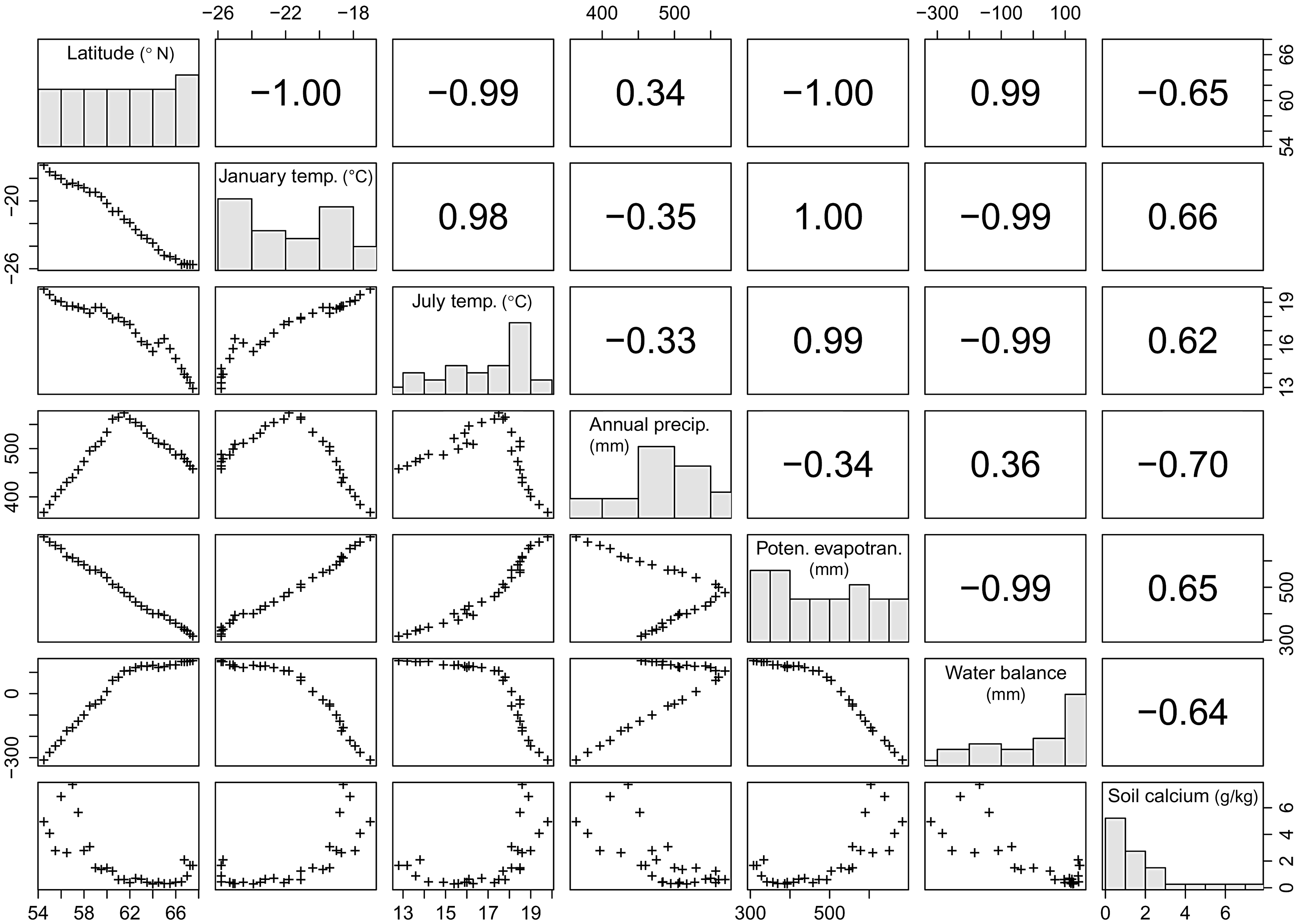

Supplement: Figure S1 — Patterns of selected climatic and environmental variables along the studied latitudinal transect and their pairwise relationships. The upper right part shows values of Spearman correlation coefficient. (TIF) [file pone.0104035.s001.tif]

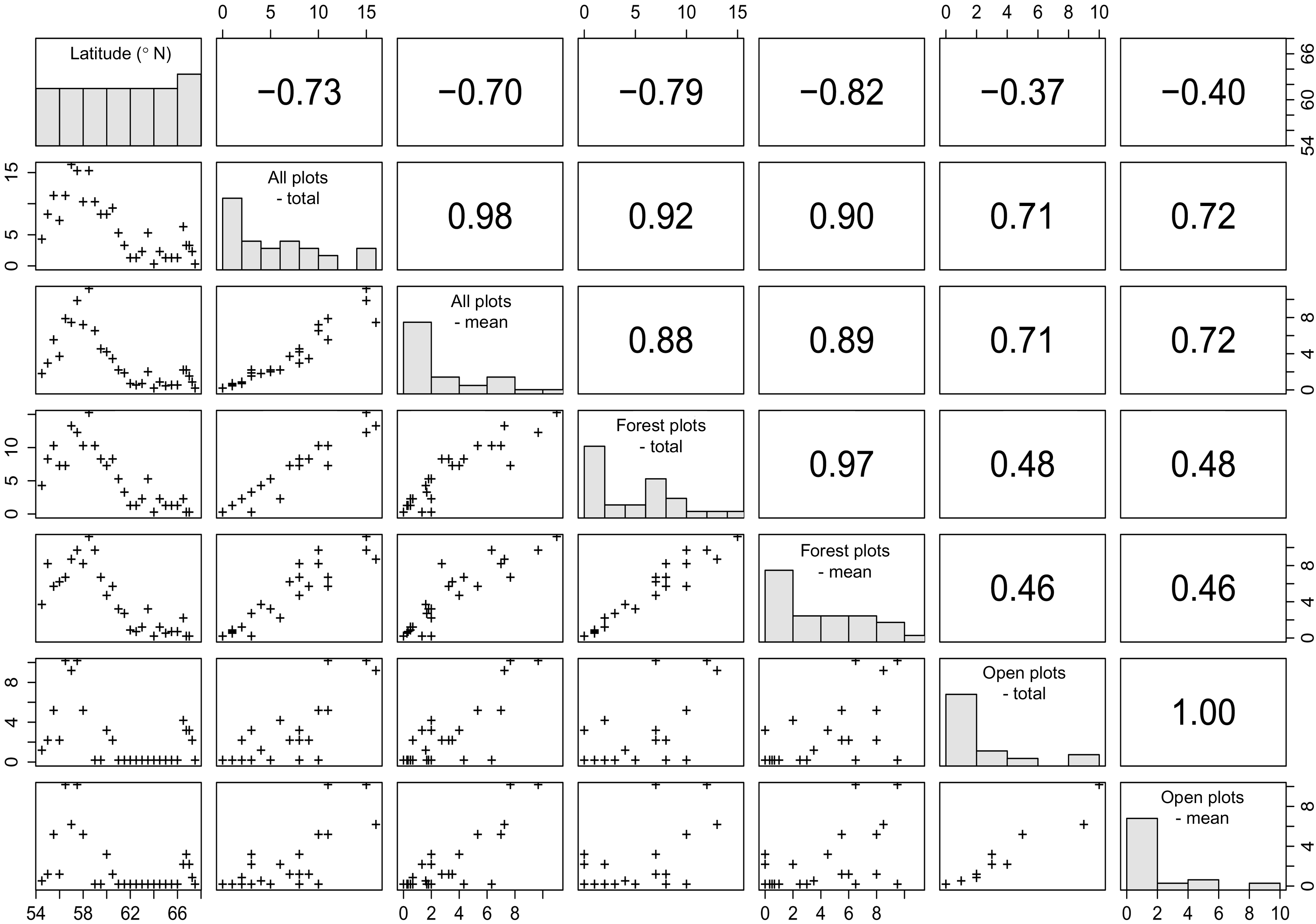

Supplement: Figure S2 — Changes in land-snail species numbers measured in different habitats and different ways along the studied latitudinal transect and their pairwise relationships. The upper right part shows values of Spearman correlation coefficient. (TIF) [file pone.0104035.s002.tif]
